# Development and Application of a Hybrid Support of Laccase from *Trametes versicolor* with Zinc Oxide Nanoparticles for Textile Dye Degradation

**DOI:** 10.1021/acsomega.5c08988

**Published:** 2026-01-28

**Authors:** Sabrina Grando Cordeiro, Ani Caroline Weber, Guilherme Schwingel Henn, Jéssica Samara Herek dos Santos, Ana Laura da Rocha, Caroline Schmitz, Elziane Pereira Ferro, Daniel Kuhn, Valeriano Antônio Corbellini, Odorico Konrad, Eduardo Miranda Ethur, Lucélia Hoehne

**Affiliations:** † University of Vale do Taquari—Univates, Lajeado, Rio Grande do Sul ZC 95914-014, Brazil; ‡ 67889 University of Santa Cruz do Sul—UNISC, Santa Cruz do Sul, Rio Grande do Sul ZC 96815-900, Brazil

## Abstract

A hybrid support based on sodium alginate and green-synthesized ZnO nanoparticles was developed for the immobilization of laccase (*Trametes versicolor*) and applied to textile dye degradation. ZnO nanoparticles were biosynthesized using pecan shell (*Carya illinoinensis*) extract in combination with a zing precursor salt (Zn­(NO_3_)_2_·6H_2_O) and characterized by ultraviolet–visible (UV–Vis) absorption spectroscopy, Fourier transform infrared spectroscopy with attenuated total reflectance (FTIR–AR), scanning electron microscopy (SEM), and energy-dispersive X-ray spectroscopy (EDS), confirming their structural and morphological features. Laccase was entrapped in sodium alginate beads and cross-linked with divalent cations (0.2 M CaCl_2_, BaCl_2_ or CuSO_4_) supplemented with glutaraldehyde (100 μL of 50% v/v) during bead formation; beads were kept in the cross-linking solution for 60 min, washed and stored at 4 °C. Immobilized systems were evaluated for immobilization yield, efficiency, enzyme leakage, and storage stability. Among the tested agents, Cu^2+^ provided the highest immobilization efficiency (52%) with reduced enzyme leakage. The hybrid support containing ZnO nanoparticles (Alg + ZnO-NPs@Lac) exhibited remarkable improvements in enzyme stability, maintaining 153% of its initial activity after 35 days, compared to only 68% for alginate without ZnO. The immobilized biocatalyst demonstrated enhanced tolerance to pH and temperature variations and achieved up to 86% degradation of Reactive Blue 198 under optimized conditions. These findings highlight the synergistic effect of ZnO nanoparticles in strengthening the mechanical and thermal resistance of the alginate matrix and improving laccase stability, offering a sustainable and efficient platform for the enzymatic treatment of textile dyes and emerging micropollutants in wastewater.

## Graphical abstract

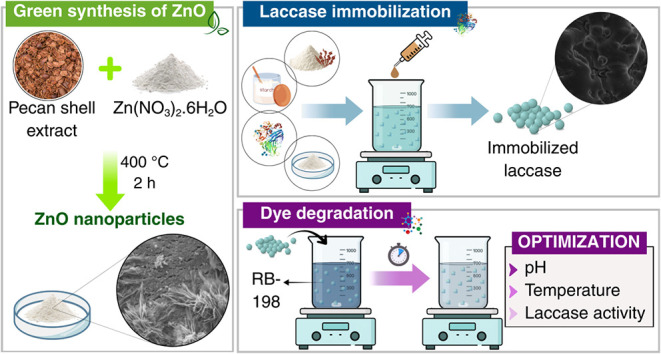

## 1 Introduction

Chemical contamination, especially wastewater generated by the textile industries, contains a significant number of dyes and toxic synthetic mixtures that contribute to environmental toxicity.^1^ Depending on their concentration, these compounds may be classified as micropollutants.^2^ Consequently, there is an urgent need to develop methodologies capable of decomposing such contaminants in nature, since conventional systems are not able to achieve this.^3,4^ Among the most applied methods are physical processes (adsorption and membranes)^4^ and chemical processes (photo-oxidation, photocatalysis, and electrochemistry).^5^ However, most of these approaches are either costly to apply or tend to generate toxic byproducts.^6^ Therefore, there is a growing demand for technologies that combine high efficiency with low environmental impact. In this context, biocatalysis has quickly emerged as a tool of great interest in both science and industry, as reflected in the increasing market interest in enzymes.^7,8^

Laccases, due to their high oxidative capacity, have been investigated as a potential alternative to conventional methods for the degradation of emerging pollutants.^9^ Despite the considerable potential of enzymatic processes in industrial applications, they still present certain drawbacks, such as rapid loss of enzymatic activity, difficulties in recovery and reuse of the biocatalyst, and low stability under different operational conditions.^9,10^

Enzyme immobilization has been reported as an effective strategy to address several limitations associated with free enzymes, as it generally provides a more stable biocatalyst with higher activity, as well as recovery and reuse potential.^10–12^ The main challenge, however, lies in selecting the appropriate immobilization method and the most suitable support for each enzyme.^13^ Among the various supports, biopolymers have been widely investigated due to their low cost, nontoxicity, biocompatibility, and high immobilization capacity.^14^ More recently, hybrid materials have been developed to improve the mechanical and thermal properties of biopolymers. According to IUPAC, hybrid materials are defined as composites formed by the combination of organic and inorganic compounds.^15,16^

Polysaccharides such as alginate have been extensively applied in this context, often in combination with metal oxides and magnetic nanoparticles. These approaches have been associated with improved immobilization efficiency, easier separation from the medium, and enhanced mechanical and thermal resistance of alginate-based supports.^14^ The synthesis route of nanomaterials is also critical, as it directly affects their biocompatibility with both the enzyme and the biopolymer. In line with the principles of environmentally friendly technologies such as biocatalysis, green chemistry strategies have been increasingly adopted for nanomaterial synthesis.^17^

Biosynthesis has emerged as a sustainable, safer, and biocompatible alternative for nanomaterial production. Plant extracts rich in phytochemicals have been employed as mediators in the synthesis of nanometals.^18,19^ Based on this approach, the present study aims to develop a novel hybrid support for laccase immobilization, consisting of a biopolymer combined with zinc oxide (ZnO) nanoparticles biosynthesized from pecan nutshells (*Carya illinoinensis*), for the subsequent degradation of the dye Reactive Blue 198.

## 2 Results and Discussion

### 2.1 Synthesis of ZnO Nanoparticles by Green Chemistry

Pecan nuts (*Carya illinoinensis*) has a high consumption rate due to their nutritional value and the presence of antioxidant compounds.^20^ However, a significant generation of byproducts is observed, since the shell accounts for approximately 49% of the total mass of the nut.^21^ Nevertheless, studies have reported that these shells may contain relevant fractions of phenolic and antioxidant compounds, in some cases at concentrations even higher than those found in the pecan kernel.^22^ Given the wide diversity of phytochemicals present in *C. illinoinensis* shells, it is suggested that this byproduct has the potential to act as a mediating agent in the phytosynthesis of zinc oxide nanoparticles, contributing to the valorization of agro-industrial residues.^22^

The synthesis of nanoparticles (NPs) through green chemistry approaches employs phytochemicals from plants as reducing agents of metal ions (obtained from the precursor salt), leading to the formation of small metallic clusters, and stabilizing agents, which prevent excessive particle growth. In this context, the present study evaluated the effect of different concentrations of pecan shell extract on the synthesis of ZnO-NPs, considering the variation in phytochemical availability in the reaction medium.

The absorption spectra of the NPs were obtained using UV–vis spectrophotometry of suspensions prepared in 96.6° GL ethanol (0.1% w/v) (Figure 1a). In all conditions evaluated, an absorption peak at low wavelengths was observed, which is characteristic of ZnO nanoparticles.^23^ Moreover, a progressive decrease in the absorption wavelength was verified as the extract concentration increased. Based on these results, the band gap of the NPs was estimated using the Tauc method, applying the equation α*h*ν = *A*(*h*ν – *E*_g_)^n^, where α corresponds to the absorption coefficient, *h* is Planck’s constant, ν the photon frequency, *A* the proportionality constant, and *E*_g_ the material band gap.

**Figure 1:**
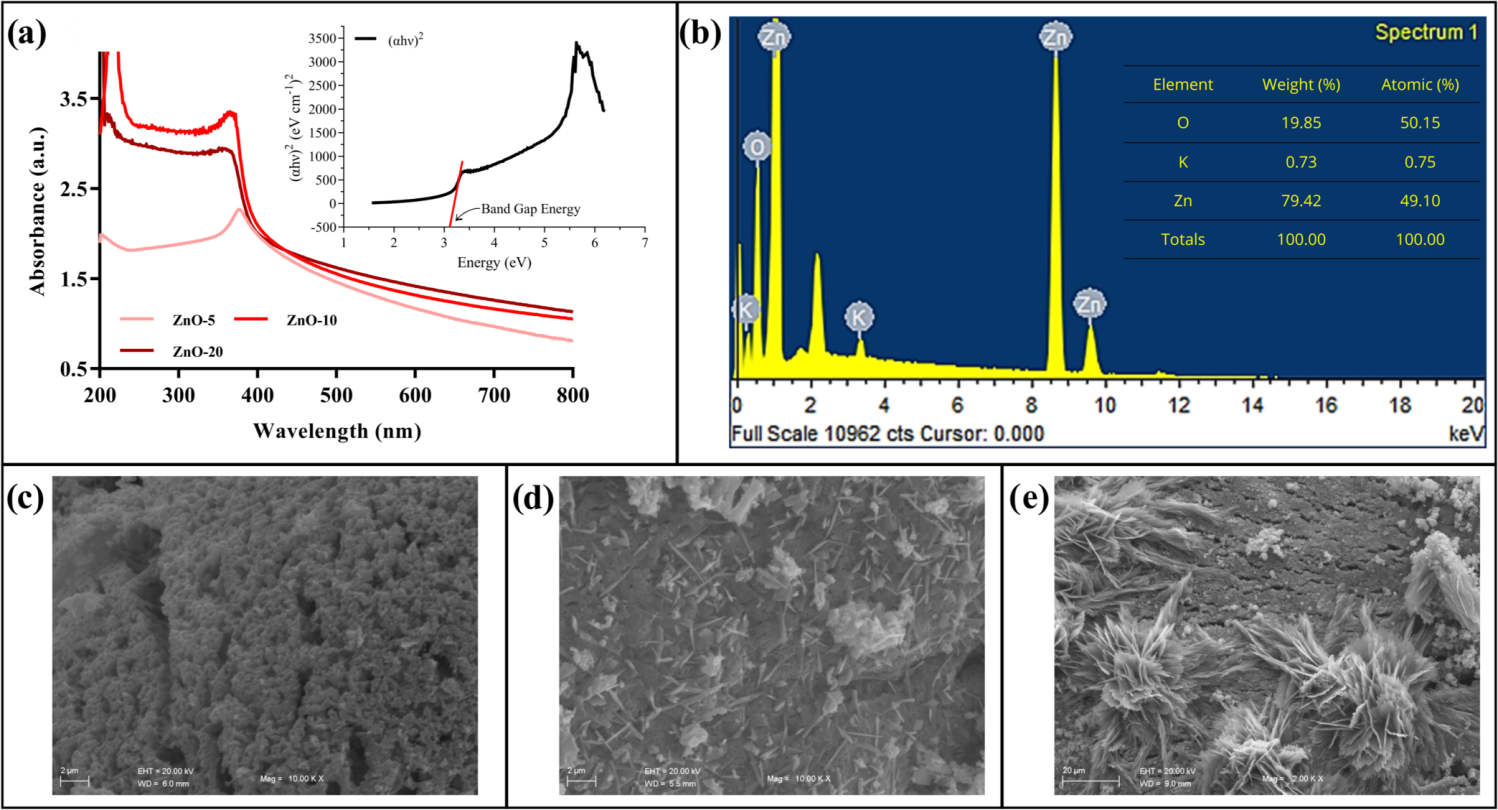
Characterization of ZnO-NPs: (A) UV–vis absorption spectrum, (B) EDS spectrum with elemental distribution, and SEM micrographs (×10,000) for (C) ZnO-5, (D) ZnO-10, and (E) ZnO-20.

The estimated band gap values of the biosynthesized ZnO-NPs increased with extract concentration, reaching 3.28, 3.97, and 3.53 eV for 5, 10, and 20 g L^–1^, respectively. These findings indicate that increasing extract concentration promotes a reduction in the average crystallite size, since smaller particles contain fewer atoms, resulting in a greater energy separation (band gap) between the valence and conduction bands.^24^

SEM morphological analyses revealed the formation of distinct ZnO-NP structures depending on extract concentration (Figure 1c–e). ZnO-5 (Figure 1c) exhibited a nebulous structure with small particle aggregates, while ZnO-10 (Figure 1d) showed irregular rod-shaped crystals. In turn, ZnO-20 (Figure 1e) displayed tridimensional flower-like structures. These results demonstrate that extract concentration exerts a direct influence on the morphology of the synthesized NPs.

The EDS spectrum (Figure 1b) showed a predominance of oxygen, followed by zinc, in addition to vestigial amounts of potassium, possibly related to the plant extract used in biosynthesis. Considering these findings, ZnO-20 nanoparticles (synthesized with 20 g L^–1^ pecan shell extract) were selected for the formulation of the hybrid support Alg + ZnO-NPs@Lac, due to their tridimensional morphology, which favors enzyme immobilization when compared to nebulous or rod-like structures.

### 2.2 Morphological Characterization of the Hybrid Support Alginate+ZnO-NPs@Lac

The morphological features of the hybrid support with immobilized laccase were evaluated using scanning electron microscopy coupled with energy-dispersive X-ray spectroscopy (SEM-EDS) (Figure 2a,b). For comparison, a control sample of Alg + Lac (Figure 2c,d), without ZnO-NPs, was also analyzed, enabling the identification of differences in both the alginate bead structure and the elemental composition of the materials. In the Alg + ZnO-NPs@Lac structure, small crystals were observed on the surface of the alginate matrix, most likely corresponding to ZnO incorporated into the support. This assumption was confirmed by the elemental distribution obtained through EDS, which revealed the presence of carbon and oxygen, attributed to alginate; sulfur and copper, derived from the cross-linking agent (CuSO_4_); and zinc, originating from the nanoparticles. In contrast, the control Alg + Lac showed no Zn in its composition and exhibited a more homogeneous structure, without surface crystals.

**Figure 2:**
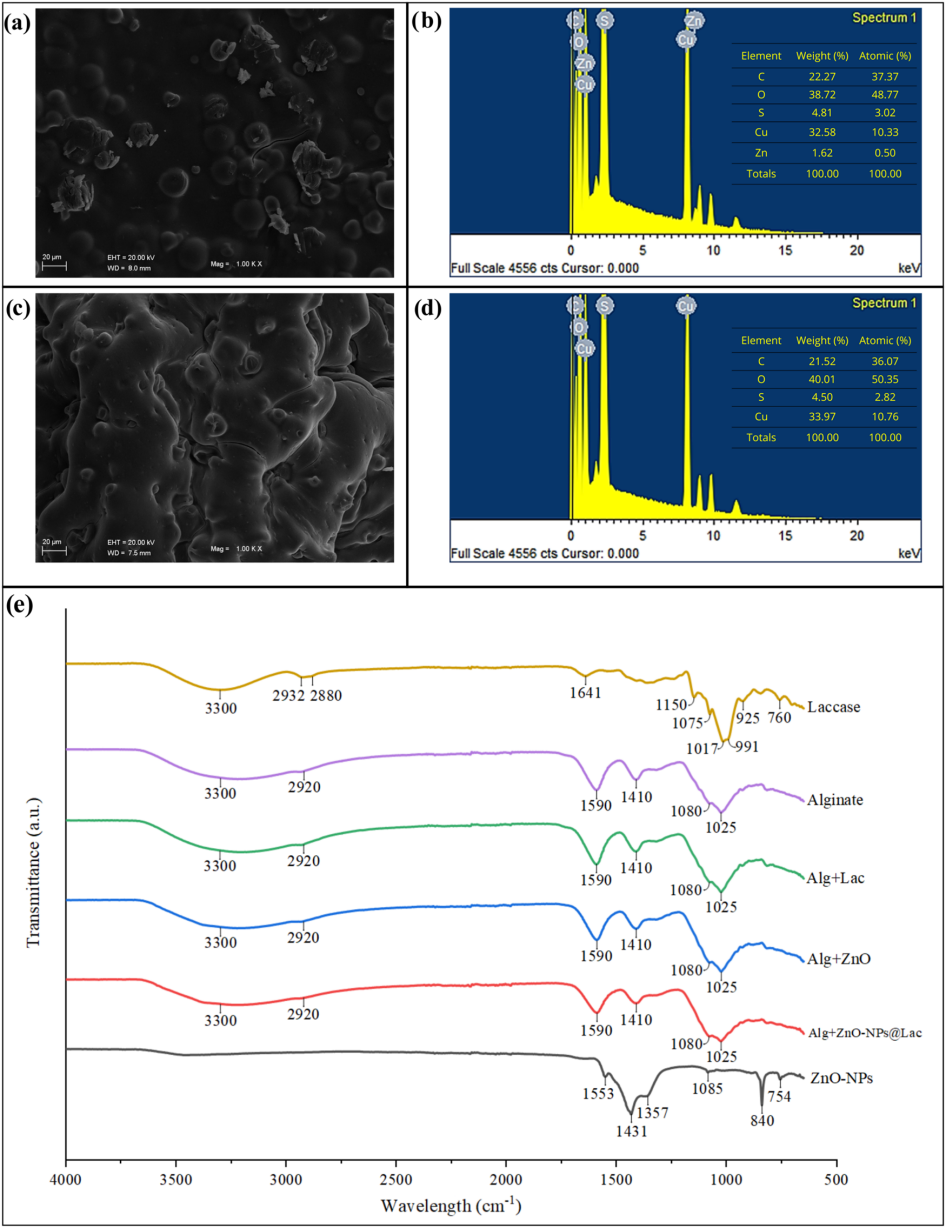
Characterization of supports containing immobilized laccase. SEM micrographs (×1000) with EDS spectra for (a and b) Alg + ZnO-NPs@Lac and (c and d) Alg + Lac; (e) FTIR spectra of the supports and respective process controls.

The FTIR spectrum confirms the incorporation of laccase and ZnO nanoparticles into the alginate support (Figure 2e). The main bands observed in the spectrum of pure alginate—O–H stretching (∼3300–3400 cm^–1^), C–H stretching (∼2920 cm^–1^), COO^–^ groups (∼1600 and ∼1410 cm^–1^), and the C–O–C band (∼1020–1080 cm^–1^) remain present in the Alg + Lac sample, evidencing the preservation of the polysaccharide structural integrity. The appearance of characteristic amide bands (amide I/II, approximately between 1650 and 1540 cm^–1^) confirms the immobilization of the enzyme associated with ZnO-NPs.^25^

In the hybrid support Alg + ZnO-NPs@Lac, shifts observed in the COO^–^ bands indicate interactions between the alginate carboxylate groups, Cu^2+^ ions, and ZnO nanoparticles. These findings demonstrate the structural integration of the components, in agreement with previous studies reporting the effectiveness of hybrid support in enzyme immobilization.^25^

### 2.3 Immobilization of Laccase Using Different Cross-Linking Agents

The use of different cross-linking agents in the formation of the alginate matrix for laccase immobilization was evaluated. Table 1 presents the results of yield (%), efficiency (%), and protein leakage (%) into the storage medium after 10 days for the three cross-linking agents tested: BaCl_2_, CaCl_2_, and CuSO_4_. In addition, Figure 3 shows the stability of the Alg+Lac support over 15 days.

**Figure 3:**
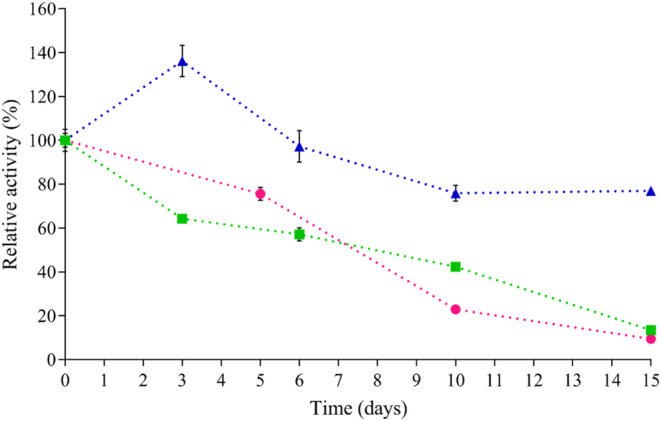
Stability of the enzyme immobilized in alginate cross-linked with CaCl_2_ (Green Box Solid), CuSO_4_ (Blue Triangle Sold), and BaCl_2_ (Pink Circle Solid) over 15 days. Each point corresponds to the mean ± standard deviation of three independent experiments performed in triplicate.

**Table 1:** Immobilization Yield and Efficiency, and Enzyme Leakage Into the Storage Medium According to the Cross-Linking Agent Used^a^

cross-linking agent	yield (%)	efficiency (%)	leakage after 10 days (%)
CaCl_2_	96.0 ± 1.00^ab^	12.0 ± 0.01^b^	57.7 ± 0.68^b^
CuSO_4_	94.9 ± 1.47^b^	52.0 ± 3.36^a^	24.1 ± 0.10^a^
BaCl_2_	98.1 ± 0.83^a^	5.3 ± 5.68^b^	77.1 ± 0.02^b^

a Mean ± standard deviation. Different letters within the same column indicate a statistically significant difference (*p* < 0.05).

The results in Table 1 indicate that the immobilization yield of laccase in the alginate support was satisfactory, with no major variation among the tested conditions, although slightly higher values were achieved with Ba^2+^ and Ca^2+^ compared to Cu^2+^. However, the markedly higher immobilization efficiency and stability observed for Cu^2+^–alginate system can be attributed to the stronger coordination of Cu^2+^ ions with both the alginate matrix and the enzyme surface, which reinforces the structural network and minimizes protein leakage during storage.^12,41^ On the other hand, when immobilization efficiency was evaluated, which reflects the retention of catalytic activity of the trapped protein, significant differences were observed among the conditions. Immobilization using copper as the cross-linking agent resulted in the highest values, with immobilization efficiency of 52.0% and protein leakage after 10 days corresponding to 24.1% of the initial activity. In contrast, supports cross-linked with Ba^2+^ and Ca^2+^ exhibited low immobilization efficiencies (5.3% and 12.0%, respectively), and rapidly lost activity due to enzyme leakage.

Alginate is a linear polymer composed of guluronic (G) and mannuronic (M) acids, which has the ability to form stable gels in the presence of multivalent cations. Gel formation occurs due to the interaction of these cations with the hydroxyl and carboxyl groups of G units, leading to cross-linking between polymer chains and the formation of a tridimensional structure, resulting in the alginate gel^26^ (Figure S1). Although Ca^2+^ is the most used cation due to its biocompatibility with biological materials, other ions exhibit greater affinity for the matrix. According to the literature, the affinity of alginate for divalent ions follows the order: Pb^2+^ > Cu^2+^ > Cd^2+^ > Ba^2+^ > Sr^2+^ > Ca^2+^ > Co^2+^ > Ni^2+^ > Zn^2+^ > Mn^2+^.^27,28^

Among the three cross-linking agents evaluated, Cu^2+^–alginate exhibited superior performance, maintaining higher enzymatic activity throughout the 15-day storage period compared to Ca^2+^– and Ba^2+^–alginate (Figure 3). Specifically, the Cu^2+^–cross-linked system retained approximately 77% of its initial activity after 15 days, whereas Ca^2+^– and Ba^2+^–based supports retained less than 15%. This result indicates that copper ions promote the formation of more stable and efficient matrices. It should be noted that the sampling intervals in the stability assays were not strictly identical for all immobilized systems at the initial time points; however, all samples were stored under the same experimental conditions (temperature, pH, enzyme concentration, and medium volume), ensuring that the observed differences reflect intrinsic stability rather than experimental variability. The activity decay profiles were highly consistent and reproducible across replicates, and the magnitude of the differences between systems was sufficiently large to allow a reliable qualitative and comparative assessment of stability trends.

Although calcium ions are commonly employed for enzyme immobilization, copper-based alginate gels have been widely used for laccases. This preference is likely associated with the nature of laccase as a multicopper oxidase (Figure S2), whose catalytic mechanism relies on the presence of copper atoms within its structure. Thus, the structural compatibility between the enzyme and the Cu^2+^ ions in the support contribute to maintaining its catalytic activity and overall stability. Moreover, Cu^2+^ ions display strong affinity for alginates, forming stable complexes with the polymeric network that enhance the mechanical strength of the beads. Calcium alginate spheres are typically more porous and chemically less stable than those cross-linked with copper, which often leads to higher enzyme leakage.^30^

Additionally, previous studies indicate that the counterions Cl^–^ from BaCl_2_ and CaCl_2_ may act as inhibitors of laccase activity by interacting with the T2 and T3 copper sites, thereby blocking electron transfer from the T1 site and impairing catalysis (Figure S2). However, this inhibition is reversible and competitive, which does not entirely preclude the use of CaCl_2_.^29,30^ Within alginate matrices, Ca^2+^ and Ba^2+^ can coordinate with carboxylate groups (−COO^–^), potentially displacing Cu^2+^ from active sites or altering the geometry of T2/T3 centers. Conversely, Cu^2+^ maintains structural compatibility with these sites, preserving enzymatic activity. Similar trends have been reported in other works, reinforcing the conclusion that Cu^2+^–alginate provides a more effective matrix for laccase immobilization compared to calcium or barium ions.^31–33^ Nevertheless, the influence of additional factors, such as support microstructure or local diffusion effects, cannot be entirely ruled out. Based on these findings, Cu^2+^ was selected as the cross-linking agent for subsequent experiments.

### 3.4 Immobilization of Laccase in Hybrid Alginate and ZnO-NPs Support

The immobilization percentages of laccase on Cu-alginate and on the hybrid support containing ZnO nanoparticles were also investigated in this study. The results of yield, efficiency, and stability for both immobilization strategies are presented in Table 2.

**Table 2:** Yield, Efficiency, and Stability of Laccase Immobilized on Cu-Alginate and on the Hybrid Support Containing ZnO-NPs^a^

immobilization condition	yield (%)	efficiency (%)	stability after 35 days (%)
Alg + Lac	94.9 ± 1.46^a^	52.0 ± 3.36^a^	68.33 ± 0.24^b^
Alg + ZnoNPs@Lac	96.8 ± 0.27^a^	37.3 ± 0.31^b^	153.48 ± 0.21^a^

a Mean ± standard deviation. Different superscript lowercase letters in the same column indicate a statistically significant difference (*p* < 0.05) for the parameter evaluated (yield, efficiency or stability).

When comparing the values, no statistically significant difference was observed in immobilization yield between the two supports, both around 95%. However, a considerable difference was found regarding immobilization efficiency, which reflects the retention of enzymatic activity after the process. Immobilization in the hybrid support (Alg + ZnO-NPs@Lac), due to the presence of NPs, may have reduced the pore size of the Cu-alginate matrix, hindering the substrate’s diffusion into the bead interior and the release of the product.

Nevertheless, analysis of the stability profile of both supports (Figure 4) revealed that the hybrid support exhibited an increase in laccase activity during the first 5 days, reaching approximately 30% above the initial value. Moreover, this enhanced activity remained stable at around +153.5% over the entire 35-day observation period. In contrast, immobilization in the Alg + Lac support also showed an initial increase during the first 5 days but subsequently showed a decline in activity after day 15, retaining only about 68.3% of its initial activity. A similar phenomenon was reported by^34^ when immobilizing laccase in alginate and graphene oxide-doped alginate supports.

**Figure 4:**
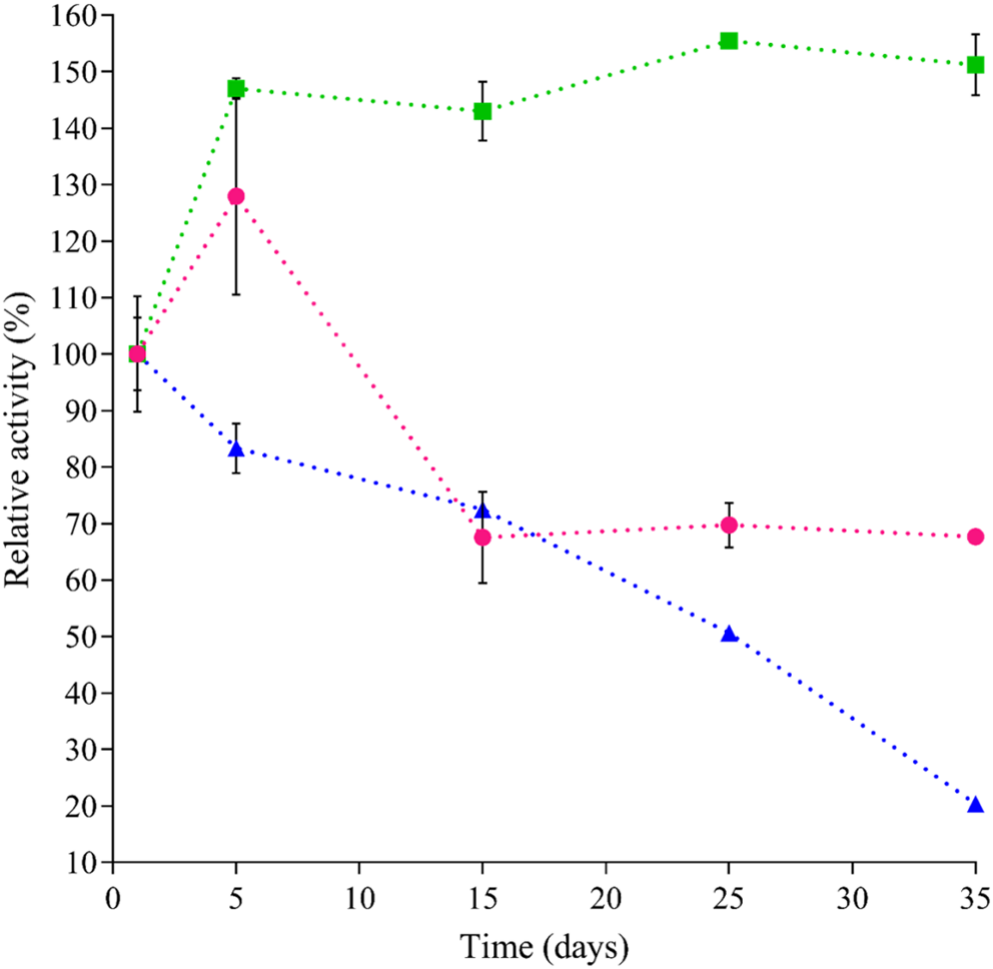
Relative enzymatic activity of (%) immobilized laccase over 35 days of storage: laccase immobilized in alginate (Alg + Lac, Pink Circle Solid), laccase immobilized in alginate containing ZnO nanoparticles (Alg + ZnO-NPs@Lac, Green Box Solid), and free laccase (Blue Triangle Solid). Each point corresponds to the mean ± standard deviation of three independent experiments performed in triplicate. Relative enzymatic activity (100%): Alg + ZnO-NPS@Lac = 0.043 U/mL; Alg + Lac = 0.060 U/mL; Free laccase = 0.271 U/mL.

In this case, the presence of ZnO within the alginate matrix can be correlated with the enhanced stability of the enzyme. This may occur due to the hydroxyl functional groups (−OH) of ZnO, which tend to interact with the functional groups of the enzyme through weak interactions, such as hydrogen bonding. These interactions do not directly affect the catalytic site but provide greater structural stability, facilitating electron transfer between the enzyme and the substrate.^34^

Additionally, as shown in Figure 4, the activity of the free enzyme, i.e., nonimmobilized and diluted in the storage medium, progressively decreased until it became almost completely inactive over the same evaluation period. This indicates that immobilization significantly enhances the storage of laccase stability by preventing its denaturation.

### 3.5 Degradation of RB-198

The ability of laccase to degrade organic compounds, either in its free form, immobilized in alginate, or in a hybrid support, was evaluated using the textile dye RB-198 as a model compound. The influence of operational variables was analyzed, including biocatalyst dose (0.01–0.6 U mL^–1^), medium pH,^2–12^ and operating temperature (25–45 °C). Figure 5 presents the obtained results, aiming to optimize the dye degradation conditions for the different enzyme forms: free, immobilized in alginate, and immobilized in the hybrid support.

**Figure 5:**
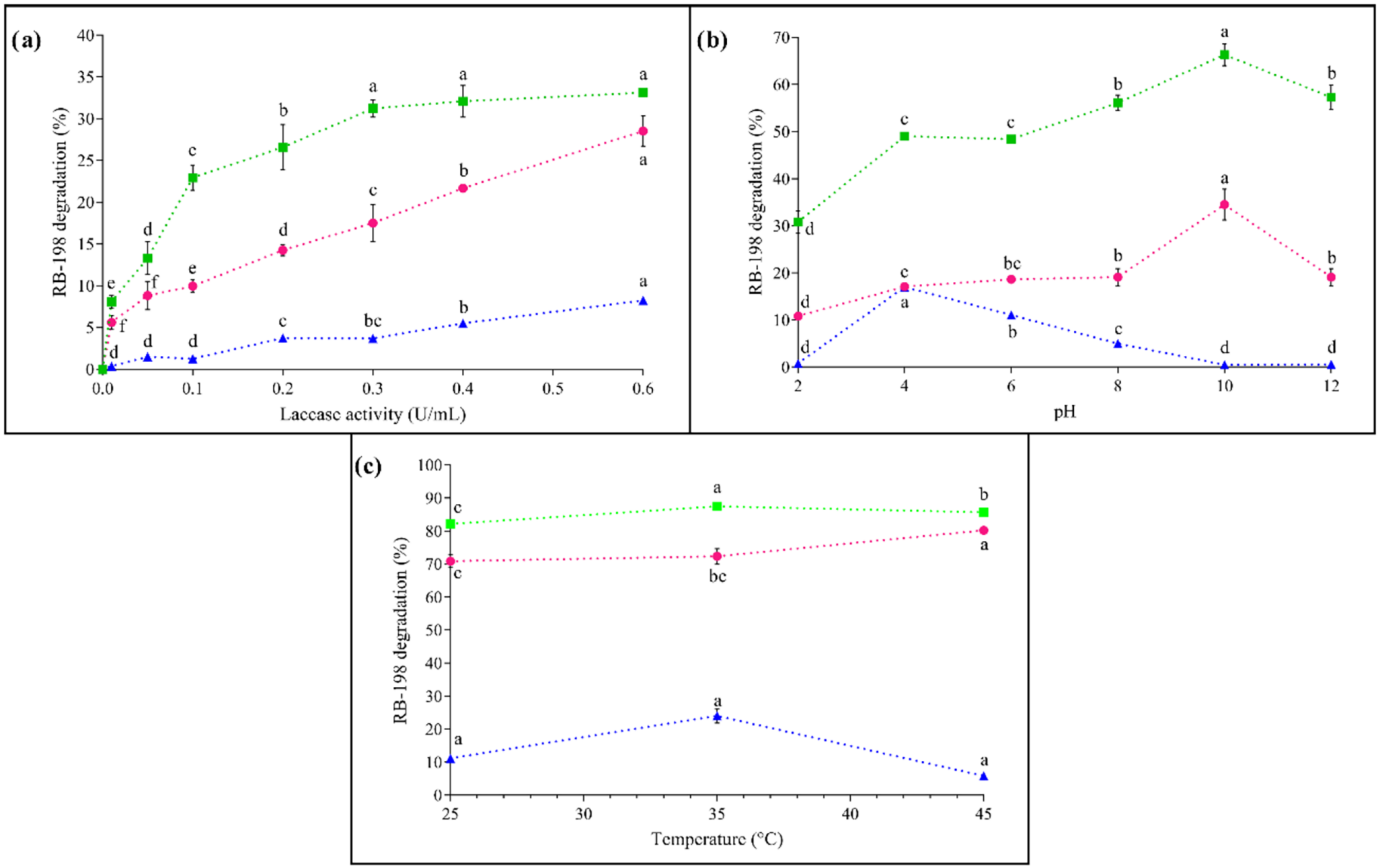
Effect of operational variables on the degradation of the textile dye RB-198: (a) laccase activity, (b) medium pH, and (c) operating temperature, using the enzyme in its free form (Blue Triangle Solid), immobilized in alginate support—Alg + Lac (Pink Circle Solid), and immobilized in the hybrid support containing ZnO-NPs —Alg + ZnO-NPs@Lac (Green Box Solid). Each point corresponds to the mean ± standard deviation of three independent experiments performed in triplicate. Different letters for the same enzyme form (free, Alg + Lac, or Alg + Zn-NPs@Lac) within a given evaluated condition (enzymatic activity, pH, or temperature) indicate a statistically significant difference (*p* < 0.05) in the percentage of RB-198 degraded.

The degradation rate of RB-198 was evaluated by varying the biocatalyst concentration in the medium at a fixed reaction time of 2 h. As shown in Figure 5a, dye removal by the Alg + ZnO-NPs@Lac system increased continuously with higher catalyst doses until equilibrium was reached at 0.03 U mL^–1^. This behavior is attributed to the greater availability of active sites in the reaction medium, which increases the probability of collisions between substrate molecules and the catalyst. However, after equilibrium, the excess of active sites no longer contributes to the reaction rate, becoming irrelevant to process efficiency.

For the controls (Alg + Lac and free laccase), a similar pattern was observed, with increasing degradation rates as a function of catalyst dose. Nevertheless, equilibrium was not reached under the tested conditions, indicating that higher doses would be required to achieve comparable degradation rates to the hybrid support. This result was expected, considering the superior enzymatic activity of the hybrid system compared to the other controls.

Solution pH is a critical factor since it can affect enzyme conformation. The highest degradation rates for immobilized laccase, both in Alg + ZnO-NPs@Lac and Alg + Lac, were observed at pH 10, whereas free laccase showed maximum performance at pH 4. Free laccase exhibited greater efficiency under slightly acidic conditions but rapidly lost its activity in alkaline pH, a behavior also reported in previous studies.^35,36^ In contrast, immobilized forms maintained higher catalytic efficiency and stability across a wider pH range (Figure 5b).

These findings support the hypothesis that immobilization enhances enzyme stability and reduces sensitivity to environmental variations. While free laccase is quickly affected by hydronium ions interacting with T1 and T2 sites, immobilization provides a protective microenvironment to the protein.^33^ According to,^37^ immobilized laccases are less likely to undergo severe conformational changes, remaining stable over a wide pH range, which facilitates their catalytic application. It is noteworthy that the Alg + ZnO-NPs@Lac system achieved the highest RB-198 removal rates at all tested pH values, evidencing not only higher stability but also synergistic effects in dye interaction.

Temperature is also a crucial parameter since higher values increase molecular agitation and promote collisions between substrate and enzyme.^38^ However, excessive heating may lead to thermal inactivation, denaturation, and loss of protein tertiary structure. Under the three tested conditions, the Alg + ZnO-NPs@Lac system maintained stable degradation at ∼86%. A similar trend was observed for Alg + Lac, which achieved maximum dye removal of 80% at 45 °C. Free laccase, however, showed greater sensitivity: efficiency increased from 11% (25 °C) to 24% (35 °C), but sharply declined to 5.8% at 45 °C, confirming its thermal instability.

## 3 Conclusion

This study established a green synthesis route for zinc oxide nanoparticles using pecan nutshells, adding value to an agro-industrial residue while adhering to the principles of sustainable chemistry. ZnO-NPs incorporated into the alginate matrix resulted in an efficient hybrid support for laccase (*Trametes versicolor*) immobilization, particularly when cross-linked with Cu^2+^ ions. The resulting biocatalyst exhibited enhanced thermal and operational stability, retaining 153% of its initial activity after 35 days, in contrast to the rapid activity loss observed for free enzyme and for support without nanoparticles. Moreover, the Alg + ZnO-NPs@Lac system achieved up to 86% degradation of the reactive dye RB-198 under optimized conditions, demonstrating superior performance across a broad pH and temperature range.

These results highlight the synergistic effect between ZnO-NPs and alginate, which enhances mechanical robustness and enzymatic stability of the matrix. The proposed strategy represents a promising alternative for the treatment of textile effluents and other emerging pollutants, combining catalytic efficiency, sustainability, and applicability in industrial water reuse processes.

## 4 Material and Methods

### 4.1 Materials

Zinc nitrate hexahydrate (Zn­(NO_3_)_2_·6H_2_O), ethanol (96.6%), sodium hydroxide (NaOH), barium chloride (BaCl_2_), citric acid, and soluble starch were purchased from Synth (Santana de Parnaíba, Brazil). Food-grade alginate was obtained from GastronomyLab (Brasília, Brazil). Copper sulfate (CuSO_4_) was supplied by Neon (Recife, Brazil), while calcium chloride (CaCl_2_) was purchased from Dinâmica (Indaiatuba, Brazil). Laccase from *T. versicolor* (63 kDa, activity determined by catechol oxidation ≥ 0.5 U/mg), 2,2′-azino-bis­(3-ethylbenzothiazoline-6-sulfonic acid) diammonium salt (ABTS), and glutaraldehyde 50% were obtained from Sigma-Aldrich (Barueri, São Paulo, Brazil).

### 4.2 Green Synthesis of ZnO

Zinc oxide nanoparticles (ZnO-NPs) were synthesized through a green synthesis approach using *C. illinoinensis* shells as a source of reducing and stabilizing agents. The plant material was supplied by Grupo Pitol (Rio Grande do Sul, Brazil), cleaned, dried, and ground in a knife mill until reaching a particle size between 48 and 65 mesh. An aqueous extract was prepared from the ground shells by decoction (15% w/v at 100 °C for 12 min), filtered, and subjected to complete solvent removal using a rotary evaporator (BÜCHI Labortechnik AG, Flawil, Switzerland), yielding a dry extract powder.^39^ This powder was then resuspended in water at pH 9 at three different concentrations (5, 10, and 20 g L^–1^) to evaluate its influence on nanoparticle synthesis and yield.

The synthesis was initiated by adding the zinc precursor salt (Zn­(NO_3_)_2_·6H_2_O, 2 g L^–1^) to the extract solutions, which were maintained under constant stirring for 2 h at 60 °C. The resulting suspension was collected and centrifuged at 3000 rpm for 10 min, followed by three successive washings with deionized water. The remaining solid was subjected to thermal treatment in a muffle furnace at 400 °C for 2 h to promote the development of the crystalline structure of the ZnO-NPs. The resulting nanoparticles were characterized by scanning electron microscopy (SEM) with energy-dispersive spectroscopy (EDS), molecular absorption spectroscopy in the ultraviolet–visible region (UV–vis), and Fourier-transform infrared spectroscopy in attenuated total reflection mode (FTIR-ATR).

### 4.3 Laccase Activity

Laccase activity was determined by spectrophotometric monitoring (UV–vis molecular absorption spectrophotometer, Thermo SD2500, São Paulo, Brazil) of the oxidation of 1 mM 2,2′-azino-bis­(3-ethylbenzothiazoline-6-sulfonic acid) diammonium salt (ABTS) in 0.1 M citrate buffer, pH 5, at 420 nm. Product formation was calculated using the molar extinction coefficient of the ABTS^+^ radical cation (ε = 36,000 M^–1^ cm^–1^), according to the equation Enzyme activity (U mL^–1^) = (ΔAbs × *V*_total_ × 1000)/(ε × *V*_sample_), where ΔAbs is the change in absorbance at λ = 420 nm per minute (min^–1^), *V*_total_ is the total reaction volume (mL), ε is the molar extinction coefficient of the ABTS^+^ radical cation (M^–1^ cm^–1^), and *V*_sample_ is the volume of enzyme added to the total reaction (mL). One unit of laccase activity (U) was defined as the amount of enzyme required to oxidize 1 μmol of substrate per minute at 25 °C.^35^

### 4.4 Laccase Immobilization

Laccase immobilization was performed via entrapment in a hybrid support composed of sodium alginate and biosynthesized ZnO-NPs. Sodium alginate (5% w/v) and starch (0.5%) were dissolved in 50 mL of ultrapure water. ZnO-NPs (0.5% w/v) and 1 mL of laccase solution (9 U, 42 mg mL^–1^) prepared in 0.1 M citrate buffer at pH 5 were added. The ZnO-NPs used corresponded to those synthesized with 20 g L^–1^ of pecan shell extract (ZnO-20), forming three-dimensional flower-like structures selected for their higher surface area and favorable architecture for enzyme immobilization. The mixture was magnetically stirred for 30 min to ensure homogeneity and then dropped, using a syringe, into a cross-linking agent solution under gentle stirring in an ice bath.^38^

The influence of different cross-linking agents on matrix formation was also evaluated. For this, laccase was immobilized in alginate-only supports without ZnO-NPs. Divalent ions Ba^2+^, Ca^2+^, and Cu^2+^, prepared from BaCl_2_, CaCl_2_, and CuSO_4_, respectively, were used at 0.2 M in ultrapure water, supplemented with 100 μL of 50% glutaraldehyde (v/v). The formed beads were maintained in the cross-linking solution for 60 min, washed with ultrapure water, and stored at 4 °C. Once the optimal cross-linking agent was identified, laccase was immobilized in the hybrid ZnO-NPs functionalized support (Alg + ZnO-NPs@Lac) under the same conditions.

Immobilization yield was calculated as Immobilization yield (%) = [(*A*_i_ – (*A*_s_ + *A*_l_))/*A*_i_] × 100, where Ai is the initial laccase activity, *A*_s_ is the activity remaining in the cross-linking solution, and *A*_l_ is the activity in the washings.^39^ Immobilization efficiency was determined as the percentage of total enzyme activity immobilized relative to the activity of the free enzyme, calculated as Immobilization efficiency (%) = (α_i_/α_l_) × 100, where αi is the activity percentage and αl is the free enzyme activity.^36^

The optimal cross-linking agent for Alginate-ZnO-NPs@Lac beads was selected based on the stability of enzymatic activity over time and the percentage of enzyme leaching into the storage medium, calculated as a relative percentage (%) in relation to the enzymatic activity at the initial time. Additionally, the stability of the enzyme immobilized on the hybrid ZnO-NPs support, on alginate-only support (Alg + Lac), and in its free form was evaluated over 35 days, with residual activity calculated relative to day 1. The morphological characteristics of the resulting biocatalysts were analyzed by SEM-EDS and FTIR-ATR. All immobilization assays were performed in triplicate on three separate days.

### 4.5 Textile Dye Degradation

The efficiency of the immobilized biocatalyst was evaluated using the reactive blue 198 (RB-198), prepared in ultrapure water at a concentration of 40 mg L^–1^. The degradation method was based on a previous study by the group, employing a batch process in a jacketed glass reactor with frequent magnetic stirring.^40^ Biocatalysis experiments were conducted using Alginate-ZnO-NPs@Lac, as well as the controls Alg + Lac and free laccase. The variables investigated were: (i) catalyst dose (0.01–0.6 U mL^–1^), (ii) medium pH,^2–12^ and (iii) temperature (25–45 °C).

To assess the effect of catalyst dose, appropriate amounts of free laccase, Alg + Lac, or Alg + ZnO-NPs@Lac were individually added to the RB-198 solution to achieve the desired enzymatic activity U mL^–1^. The optimum pH for RB-198 degradation was determined using the enzyme concentration previously optimized for Alg + ZnO-NPs@Lac, varying only the pH of the reaction medium. Subsequently, the optimum temperature for RB-198 degradation was determined using the previously established enzyme concentration and pH for Alg + ZnO-NPs@Lac, with temperature controlled by a thermostatic jacket. All reactions were conducted for 120 min. After each run, aliquots of the reaction mixture were collected, and the residual concentration of RB-198 was quantified by UV–Vis spectrophotometry after calibration curve construction. The initial dye concentration (40 mg L^–1^) was taken as 100%, and the percentage of RB-198 degradation was calculated according to

$$\mathrm{RB}-198\;\mathrm{degradation}\left(\%\right)=\frac{\left(\mathrm{initial}\;\mathrm{concentration}-\mathrm{residual}\;\mathrm{concentration}\right)}{\mathrm{initial}\;\mathrm{concentration}}\times 100$$

### 4.6 Statistical Analysis

All results obtained were subjected to analysis of variance (ANOVA) and Tukey′s test at 95% confident, using BioEstat 5.0.

## Supplementary Material



## Data Availability

Data supporting this study is included within the manuscript.

## Notes

Stages of calcium alginate formation: (a) monomeric units, (b) linkage between monomeric units forming the polymeric chain, (c) distribution of monomeric units along the polymer backbone, (d) “Egg-box” structure, (e) gelled polymeric network with Ca^2+^ ions arranged within the egg-box configuration (Figure S1); schematic representation of the trinuclear copper cluster in laccase and its interaction with alginate cross-linking ions (Figure S2) (PDF)

## Abbreviations Used

ZnO: zinc oxide
NPs: nanoparticles
UV–Vis: ultraviolet–visible spectroscopy
FTIR-ATR: Fourier-transform infrared attenuated total reflectance
SEM: scanning electron microscopy
EDS: energy-dispersive X-ray spectroscopy
ABTS: 2,2′-azino-bis­(3-ethylbenzothiazoline-6-sulfonic acid)
RB-198: reactive blue 198
U: enzyme activity unit
μL: microliter
mL: milliliter
g L^–1^: gram per liter
CaCl_2_: calcium chloride
CuSO_4_: copper sulfate
BaCl_2_: barium chloride
Alg + Lac: alginate + laccase control
Alg + ZnO-NPs@Lac: alginate + ZnO nanoparticles + laccase
T1, T2, T3: copper centers of laccase (type 1, 2 and 3)
