# Supplementary material for: Development and Application of a Hybrid Support of Laccase from Trametes versicolor with Zinc Oxide Nanoparticles for Textile Dye Degradation
Source: ACS Omega. 2026 Jan 28;11(5):7649–58. doi: 10.1021/acsomega.5c08988 (PMC12903175; doi:10.1021/acsomega.5c08988)
Supplement: Supplementary file 1 [file ao5c08988_si_001.pdf]

Development and application of a hybrid support of laccase from *Trametes versicolor* with zinc oxide nanoparticles for textile dye degradation

*Sabrina Grando Cordeiro[1], Ani Caroline Weber[1], Guilherme Schwingel Henn[1], Jéssica Samara Herek dos Santos[1], Ana Laura da Rocha[1], Caroline Schmitz[1], Elziane Pereira Ferro[1], Daniel Kuhn[1], Valeriano Antônio Corbellini[2], Odorico Konrad[1], Eduardo Miranda Ethur[1], Lucélia Hoehne[1]**

[1] University of Vale do Taquari – Univates, Lajeado, RS, Brazil, ZC 95914-014.

[2] University of Santa Cruz do Sul – UNISC, Santa Cruz do Sul, RS, Brazil, ZC 96815-900.

*Corresponding Author. Av. Avelino Tallini, 171, ZC 95914-014, Lajeado, RS, Brazil. Phone: +55 51 3714 7000. Email address: luceliah@univates.br

Figure S1 – Stages of calcium alginate formation. a) Monomeric units; b) Linkage between monomeric units forming the polymeric chain; c) Distribution of monomeric units along the polymer backbone; d) "Egg-box" structure; e) Gelled polymeric network with Ca^{2+} ions arranged within the egg-box configuration.

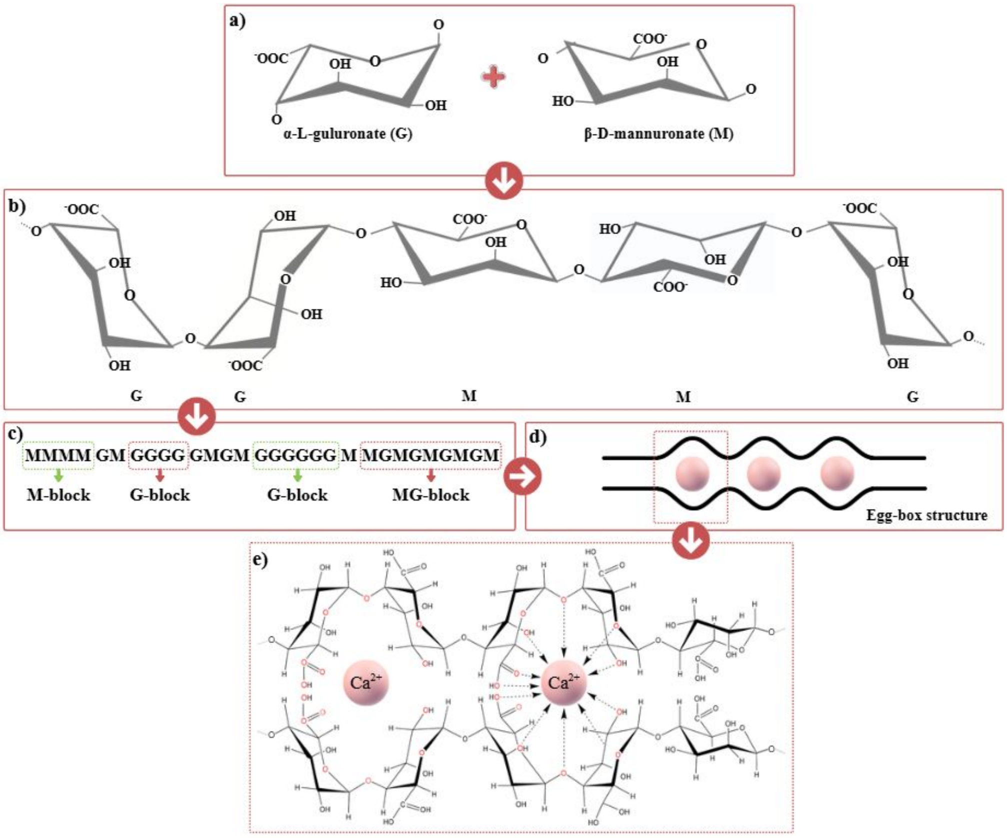

Figure S2 - Schematic representation of the trinuclear copper cluster in laccase and its interaction with alginate crosslinking ions.

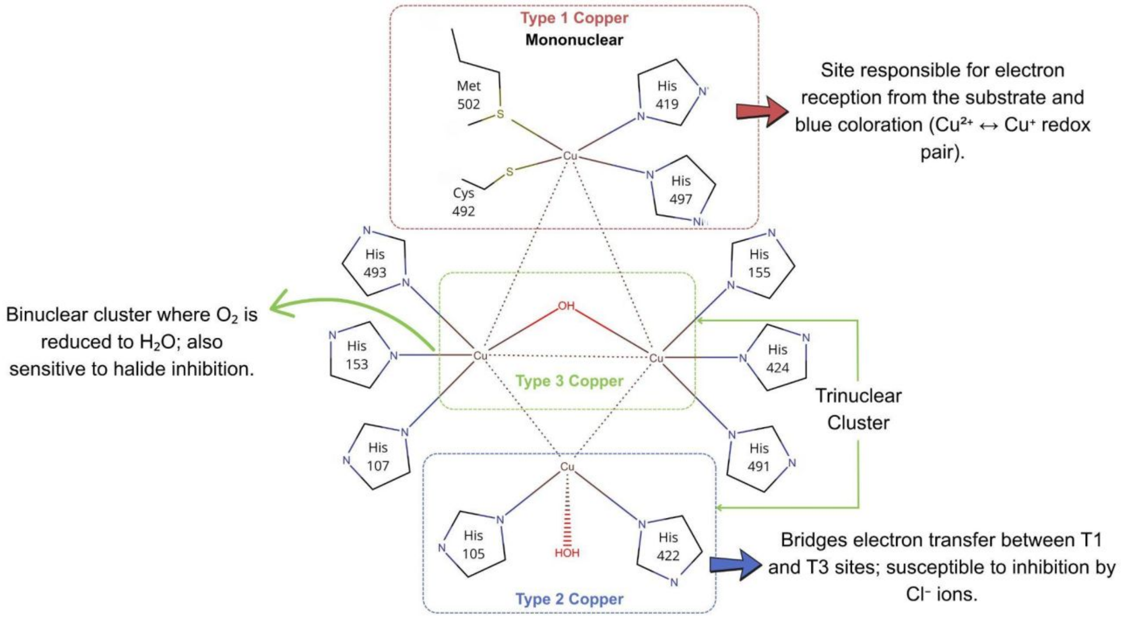

Laccase contains three types of copper centers: Type 1 (T1), a mononuclear site responsible for electron acceptance and the characteristic blue coloration; Type 2 (T2), a mononuclear site mediating internal electron transfer; and Type 3 (T3), a binuclear site that binds molecular oxygen and catalyzes its reduction to water. Together, the T2 and T3 centers form a trinuclear cluster essential for catalytic activity. In alginate-based matrices, the crosslinking ions (Cu^{2+}, Ca^{2+}, Ba^{2+}) can interact with these copper centers. Cu^{2+} exhibits both electronic and structural compatibility with the T1–T3 cluster, preserving the enzyme's functionality, whereas Cl^{-} counterions from $CaCl_2$ and $BaCl_2$ may coordinate with the T2/T3 centers, blocking electron transfer from T1 and leading to enzyme inhibition.
